# Is there a way to reduce the infection associated with external ventricular shunts? A systematic review and meta-analysis of the recent bundle of care

**DOI:** 10.1007/s10143-025-04029-4

**Published:** 2026-01-19

**Authors:** Moustapha Ramadan, Shrouk Fawze Mohamed, Ahmed A. Abdulalim, Anas Ashraf Elassal, Aya Abdelsamad, Belal Shehata, Yasmeen Farag, Mohamed Elshaer, Samar Kolkas, Nesrine Fathi Hanafi

**Affiliations:** 1 Community Medicine department, Faculty of Medicine, Alexandria University, Alexandria, Egypt; 2 Faculty of Medicine, Alexandria University Hospitals, Alexandria University, Alexandria, Egypt; 3 Neuropsychiatry resident, Faculty of Medicine, Alexandria University Hospitals, Alexandria University, Alexandria, Egypt; 4 Microbiology and Immunology Department Faculty of Medicine, Alexandria University, Alexandria, Egypt

**Keywords:** Infection, Shunt, Bundle of care, Neurosurgery, Ventricular

## Abstract

Background: Although external ventricular drains (EVDs) are crucial in neurosurgical care, they carry a high risk of healthcare-associated ventriculitis, with reported infection rates. Numerous interventions were thought to aid in infection control, but there is still an ongoing increase in infection incidence. Recently, bundled interventions with standardized protocols, antimicrobial‐impregnated catheters, tunneling techniques, chlorhexidine dressings, and limited antibiotic prophylaxis have shown an interesting efficacy in shunt-related infection control. Methods: A systematic review was conducted using PubMed, Scopus, Google Scholar, Cochrane CENTRAL, and Web of Science to search for published articles on care bundles with at least three discrete preventive measures. Data analysis was performed using R software, estimating mean difference and risk ratios. Results: Seventeen studies (9 quasi-experimental, 6 retrospective, and 2 prospective cohorts) were included in this study. Implementation of bundle care was associated with a significant reduction in the following outcomes: infection risk and drain stay duration (pooled RR = 0.32; 95% CI 0.20–0.51; *p* = 0.0001; I²=73.7%), (pooled MD=-1.57; 95% CI -5.80–2.67; *p* = 0.0038; I²=0%), respectively. Interestingly, there was no significant difference in risk with bundle of care regarding mortality (pooled RR = 1.09; 95% CI 0.62–1.92; *p* = 0.6924; I²=63.5%). However, no significant differences were observed in ventriculitis rates or hospital stay duration, with *p* = 0.0509, *p* = 0.3855, respectively. Conclusions: Bundle of care implementation significantly reduced infection risk and drain days among patients with external ventricular drains, but failed to reduce ventriculitis, mortality, and hospital stay duration. These findings support approving standardized care bundles to improve outcomes, though further high-quality research is needed to assess their impact on specific complications like ventriculitis, mortality, and hospital stay.

**Supplementary Information:**

The online version contains supplementary material available at 10.1007/s10143-025-04029-4.

## Introduction

External ventricular drains (EVDs) play a crucial role in managing serious conditions such as acute hydrocephalus, traumatic brain injury, subarachnoid hemorrhage, and various other disorders that require cerebrospinal fluid (CSF) diversion and monitoring of intracranial pressure. Besides their role in diagnosis and treatment, EVDs facilitate the sampling of cerebrospinal fluid and enable the intrathecal administration of antibiotics or chemotherapeutic agents [1, 2]. The use of EVDs carries some danger even if their intended function is vital. One of the most important problems is EVD-related infection (EVDI), which could cause meningitis or ventriculitis, far more morbidity, longer hospital stays, and more healthcare expenses [3]. 

Recent research indicated that the incidence rates of EVDI vary significantly, ranging from 2% to 45%.

This wide variation underscores the impact of differing diagnostic criteria, institutional practices, adherence to sterile technique, and patient populations on reported infection rates. Many factors contribute to such variability. Key risk factors include duration of catheterization, frequency of CSF sampling or manipulation, number of catheter insertions or changes, and presence of CSF leak or bilateral EVDs. Also, underlying pathologies — such as subarachnoid hemorrhage/intraventricular hemorrhage (SAH/IVH), immunosuppression, concomitant systemic infection, or prior neurosurgical procedures — may increase risk [4–7]. 

In consideration of these challenges, a range of preventive strategies has been suggested and implemented over time. These strategies are not limited to the implementation of antibiotic-impregnated catheters (AICs), silver-impregnated catheters, closed drainage systems, minimal handling protocols, and standardized care bundles. [8] The introduction of care bundles, which consist of a structured set of evidence-based practices designed to enhance outcomes when implemented together, has garnered significant attention. A recent meta-analysis showed that care bundles reduced the risk of EVD infections by about 54% (pooled risk ratio 0.46; 95% CI 0.33–0.65; *P* < 0.001) compared to non-bundle approaches [9]. Furthermore, meta-analyses and systematic reviews have found that both antibiotic-impregnated and silver-impregnated catheters may lower infection risk compared with standard catheters [10]. Bundles of care generally include hand hygiene practices, comprehensive barrier precautions during insertion, the implementation of AICs, ongoing staff training, and uniform protocols for CSF sampling and EVD maintenance [11]. However, reported results are highly heterogeneous across studies. Differences in study design, diagnostic criteria for infection (microbiological culture vs. clinical + laboratory criteria), definitions of catheter “duration,” frequency of sampling/manipulation, use (or not) of peri-procedural antibiotics, and types of antimicrobial coatings all vary widely. Because of this, it isn’t easy to draw definitive conclusions about the optimal bundle components or the relative efficacy of antibiotic-, silver-, or standard-impregnated catheters under different conditions (e.g., short vs. long-duration EVD) [9]. – [10].

While bundles appear effective in reducing infection rates, the data on their impact on broader outcomes such as morbidity, mortality, length of ICU/hospital stay, need for EVD replacement, and cost-effectiveness remain insufficiently characterized. This systematic review and meta-analysis aim to evaluate the safety and efficacy of the bundle of care in preventing infections and associated complications related to EVD.

## Methods

This systematic review and meta-analysis followed the guidelines of PRISMA for this study design. The protocol of this study was registered at PROSPERO, with the following ID: CRD42025648331.

### Study design and registration

This study was conducted in accordance with the Preferred Reporting Items for Systematic Reviews and Meta-Analyses (PRISMA) guidelines [12]. The study protocol was prospectively registered with PROSPERO (CRD42025648331) and is publicly available at https://www.crd.york.ac.uk/PROSPERO/view/CRD42025648331.

Approval was granted by the Ethics Committee of Alexandria University, faculty of Medicine (Date 6–7-2025/No 0307396).

### Eligibility criteria

We included randomized controlled trials (RCTs) and observational studies (cohort studies, case-control studies, and case series) that evaluated the effectiveness of a bundle of care in reducing complications associated with external ventricular drains (EVDs), particularly infections. Studies were eligible if they involved patients requiring CSF drainage, such as those with hydrocephalus or intracranial hypertension, and compared outcomes between patients managed with a care bundle versus those without or pre-protocol groups.

Primary outcomes included:

- Incidence of EVD-related infection (ventriculostomy-related infection where microorganisms mainly bacteria enter the brain through the EVD, leading to conditions like bacterial meningitis or ventriculitis.)
- Mortality rate.

Secondary outcomes included:

- Duration of EVD placement.
- Hospital length of stay.
- Microbial etiology (e.g., Gram-negative vs. Gram-positive pathogens).
- Other complications such as catheter obstruction, hemorrhage, or malposition.

### Search strategy

A comprehensive literature search was conducted in the following electronic databases: PubMed, Scopus, Web of Science, and Cochrane CENTRAL. The search strategy included terms such as “external ventricular drain,” “care bundle,” “infection prevention,” “ventriculitis,” and “CSF diversion.” No restrictions were placed on language or publication date.

### Study selection and data extraction

Two individuals assessed titles and abstracts for relevance. Full-text papers of possibly qualifying research were examined utilizing established inclusion criteria. Conflicts at any phase were settled through discussion or consultation with an additional reviewer.

Data extraction was conducted independently by two writers utilizing a standardized form. The extracted data included study information (author, year, country, design), patient demographics, specifics of the intervention and comparator, and the outcomes assessed.

### Risk of bias assessment

The Newcastle-Ottawa Scale was employed for cohort and case-control research which assessed the included papers across three domains: Selection, Comparability and outcome, whereas the JBI Critical Appraisal Checklist was utilized for quasi-experimental investigations which assessed the included studies according to several factors, including the clear establishment of the cause and effect relationship, the presence or absence of statistically significant differences among study groups, the elimination of confounding variables, the presence of a control group, the measurement of the outcome and the appropriateness of the statistical analysis used.

Each evaluation was performed separately by two reviewers, with conflicts reconciled through consensus.

### Data synthesis and statistical analysis

The meta-analysis was conducted utilizing Review Manager (RevMan) version 5.4.1. Risk ratios (RRs) with 95% confidence intervals (CIs) were computed for dichotomous outcomes. Mean differences (MDs) were employed for continuous outcomes. The I² statistic was utilized to evaluate statistical heterogeneity. In instances with significant heterogeneity (I² > 50%), a random-effects model was utilized. A fixed-effect model was employed. Sensitivity analyses were conducted by systematically excluding individual studies to evaluate the consistency of the findings. Funnel plots were generated to evaluate publication bias.

## Results

**Fig. 1:**
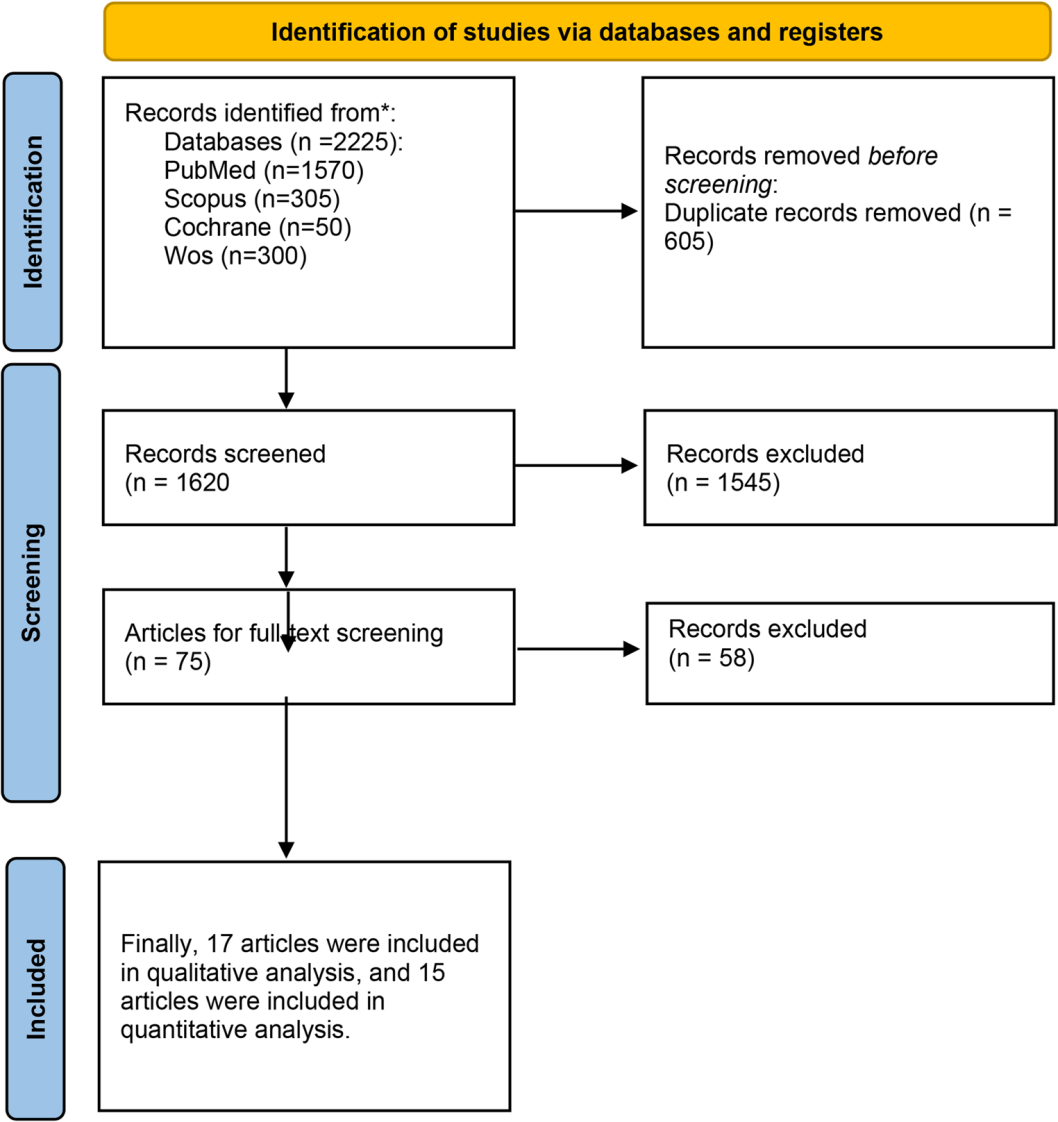
Prisma flow diagram

Literature search revealed 2225 papers through searching different databases, 605 results were duplicates, 1620 were screened by title and abstract. 1545 were excluded, and 75 were screened by full text. Finally, 17 papers were included in our study, as illustrated in Fig. 1. Out of 17 studies, 9 were quasi-experimental, 6 were retrospective, and 2 were prospective cohorts [4, 5, 13–27]. Quality assessment for the included studies showed that 9 studies were of good quality, while 3 studies were of poor quality and 5 showed fair quality, as mentioned in (Table 1) in the supplements.

Outcomes.

**Fig. 2:**
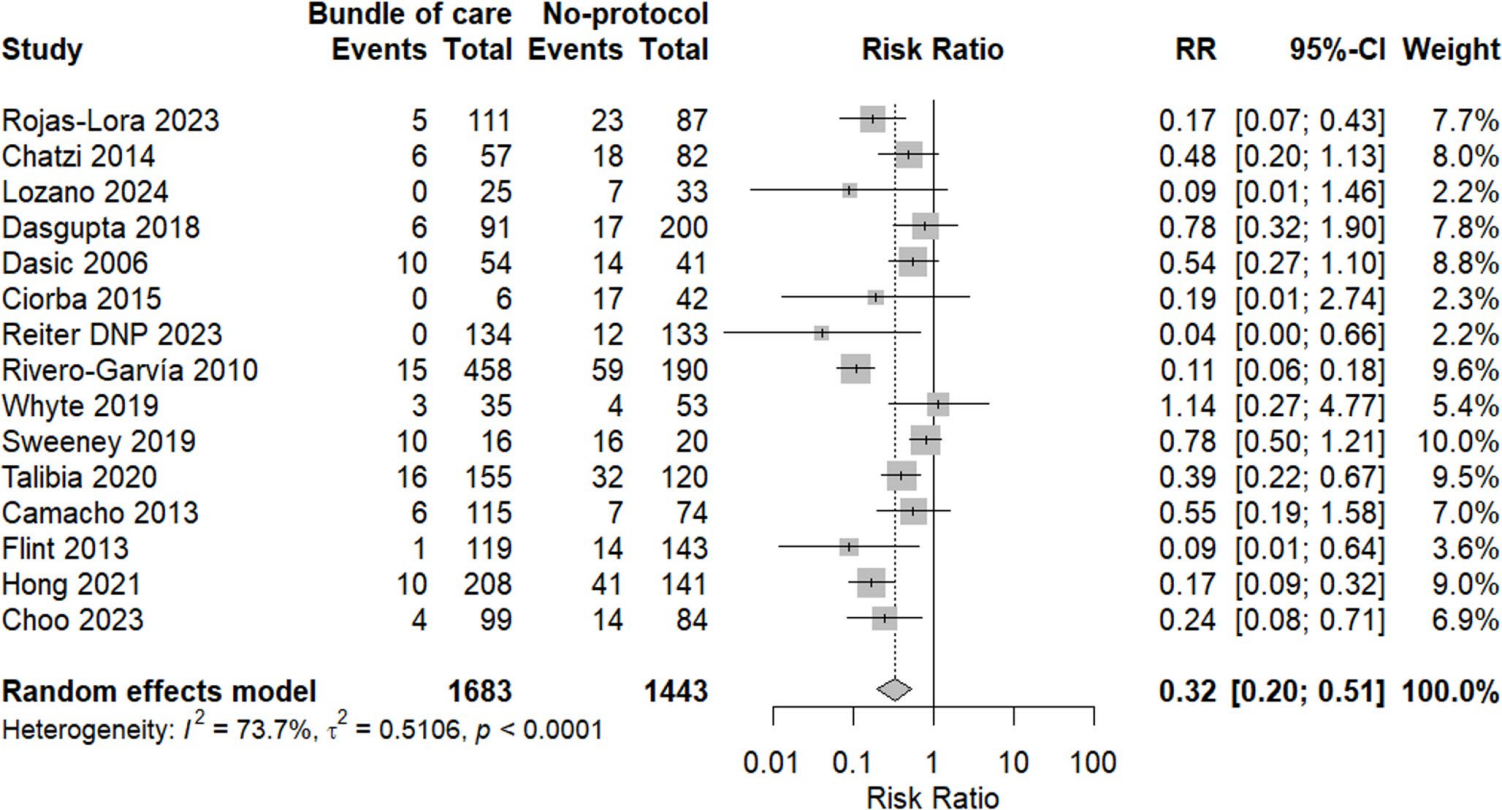
A forest plot showing the incidence of EVDI in both groups

A meta-analysis of 15 studies was done, supporting that the bundle of care showed a protective effect against EVDI in comparison to another group with an RR of 0.32 (95% CI: 0.20, 0.51, *p* = 0.0001). There was a significant heterogeneity with I^2^ = 73.7%, so a random effect model was applied as shown in Fig. 2.

**Fig. 3:**
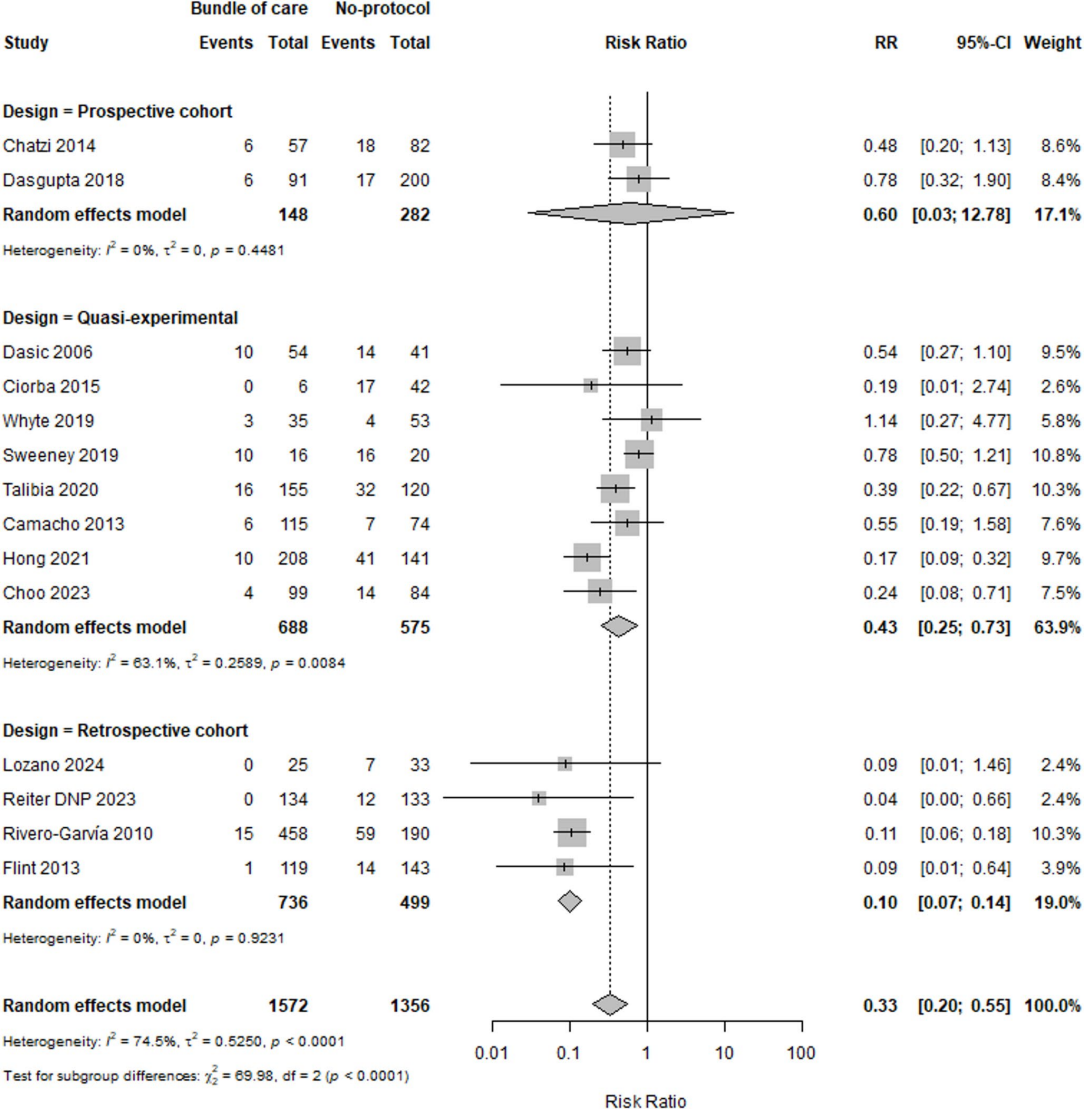
Subgrouping analysis of EVDI based on study design

Subgrouping analysis based on study design was done in a try to explore the source of heterogeneity. We found that heterogeneity was obviously resolved among retrospective cohort studies (I^2^ = 0%) without any change in the context of the results, except in prospective cohorts, where there was no significant difference between the two groups. Quasi-experimental studies showed a high level of heterogeneity, but the results remain significant (I^2^ = 63.1%). There was a significant difference between subgroups with *p* < 0.0001, as shown in Fig. 3.

**Fig. 4:**
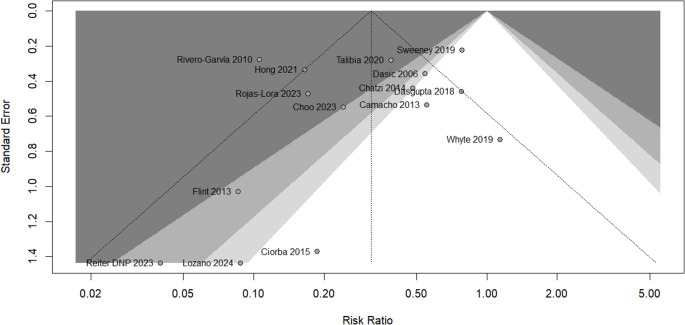
Funnel plot of studies in EVDI

Egger’s test was conducted to assess the potential presence of publication bias. We found that there was no statistically significant evidence of asymmetry in the funnel plot (*p* = 0.4324), as seen in Fig. 4.

In terms of bacterial infections, we analyzed the incidence of gram-negative and gram-negative bacteria. A meta-analysis of seven studies revealed that there was no difference between the two groups in the incidence of gram-negative (RR = 0.59, 95% CI: 0.28;1.7, *p* = 0.1442). There was a moderate reported heterogeneity, with I^2^ = 34.1%, as seen in Figure S1 in the supplements.

A sensitivity analysis was done by excluding Sweeney et al., and heterogeneity was totally resolved, with a change in the context of the results, supporting that the bundle group had a lower risk for gram-negative (RR = 0.46, 95% CI: 0.24;0.86, *p* = 0.0.025), as shown in Figure S2 in supplements.

According to the gram-positive, there was no significant difference between both groups (RR = 0.85, 95% CI: 0.32;2.21, *p* = 0.6554). Moderate heterogeneity was found (I^2^ = 53.1%), as shown in Figure S3 in the supplements.

A sensitivity analysis was done by excluding Rojas-Lora et al., and heterogeneity was reduced with I^2^ = 12.1%, with no change in the context of the results (RR = 0.67, 95% CI: 0.35;1.28, *p* = 0.1415), as shown in Figure S4 in the supplements.

**Fig. 5:**
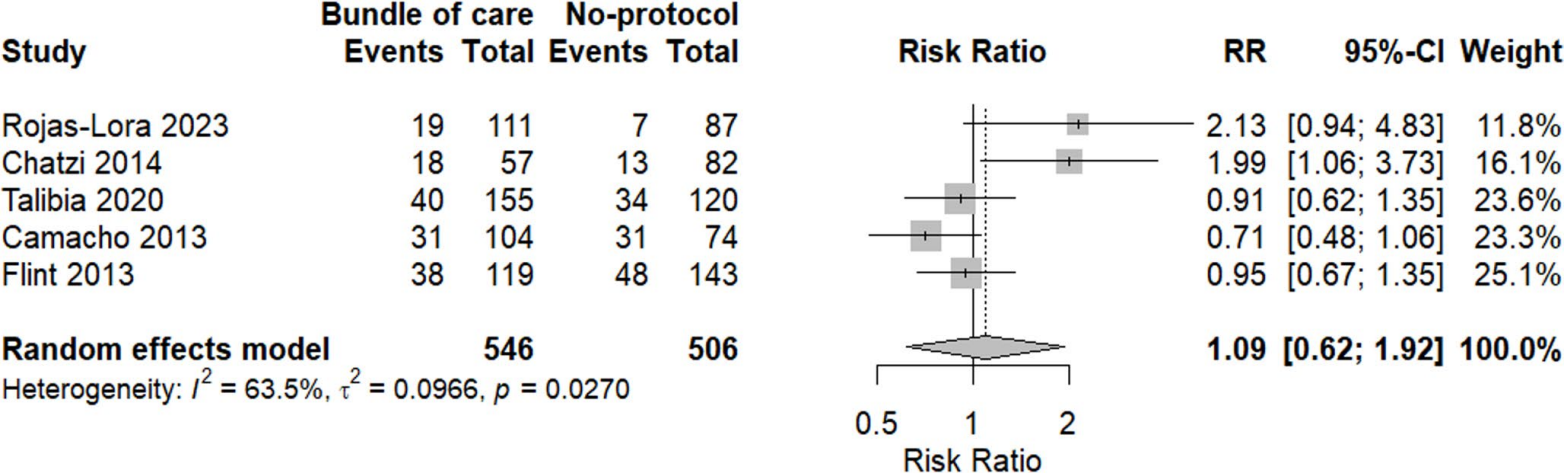
Forest plot showing mortality rates in both groups

A meta-analysis of mortality rates between the two groups was done. There was no significant difference between the bundle of care group and the other one, revealing an interesting increased risk with the bundle of care group (RR = 1.09, 95% CI: 0.62, 1.92, *p* = 0.6924). Heterogeneity was high among included studies, with I2 = 63.5%, as shown in Fig. 5.

**Fig. 6:**
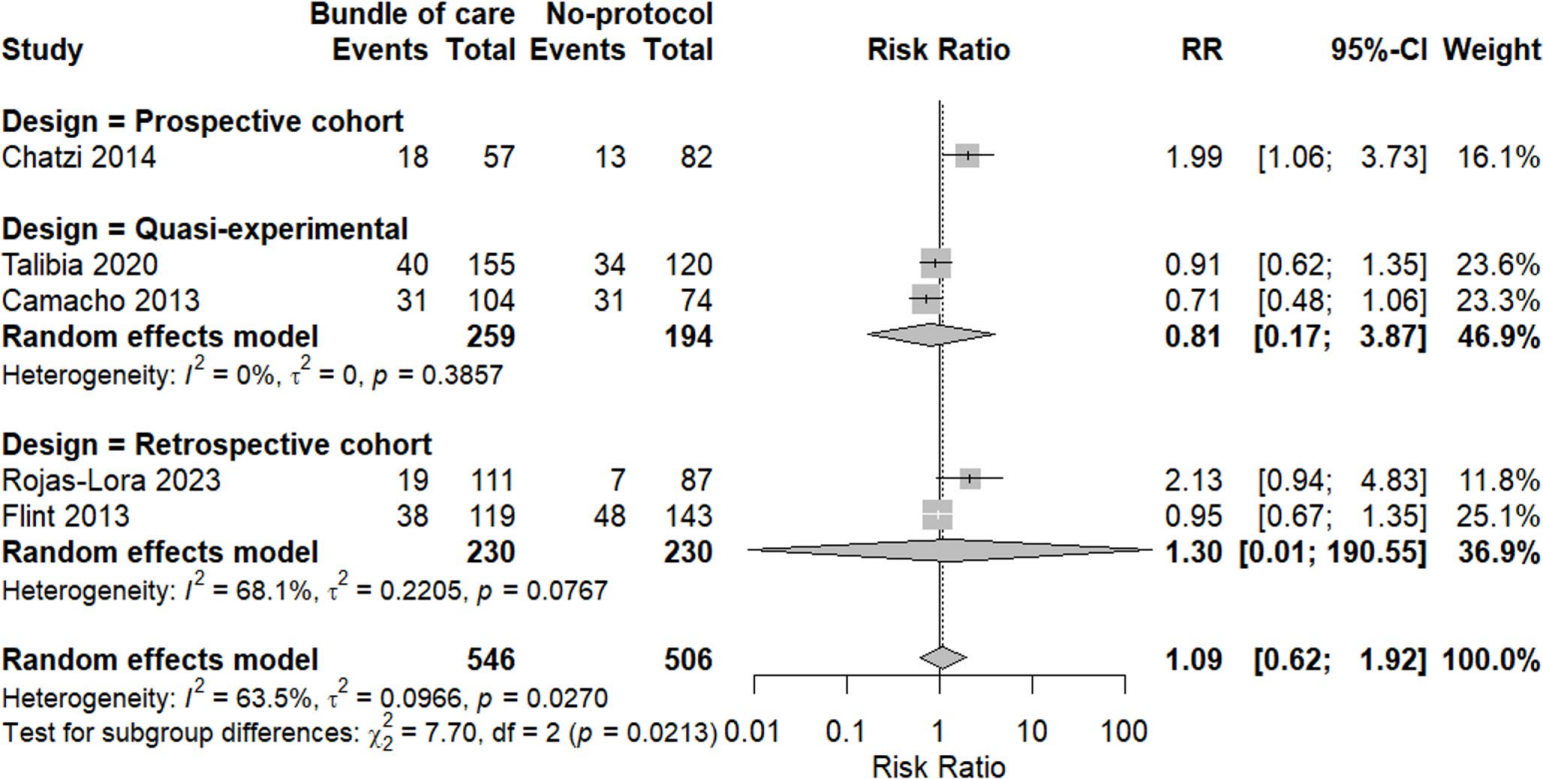
Subgrouping analysis of mortality based on study design

Subgrouping analysis was done based on study design, and we found that heterogeneity was reduced among quasi-experimental studies with I^2^ = 0%, while retrospective studies revealed moderate heterogeneity with I^2^ = 68.1%, as shown in Fig. 6.

**Fig. 7:**
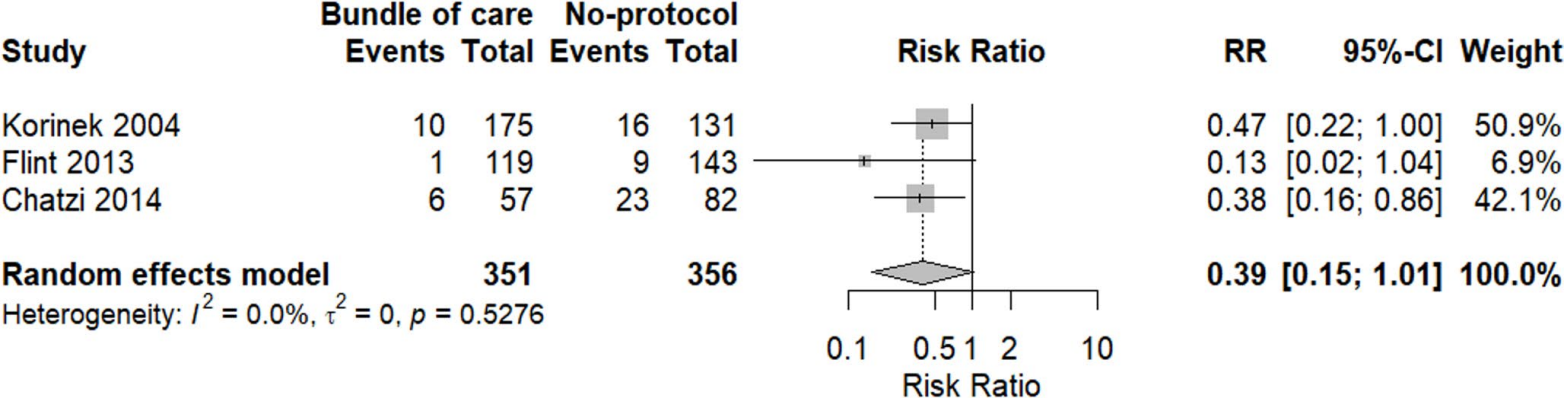
A meta-analysis of the risk of EVD-associated ventriculitis incidence between both groups

In terms of EVD-associated ventriculitis, a meta-analysis of three studies revealed that the bundle of care application did not show any significant difference in incidence of EVD-associated ventriculitis compared to the group with no protocol (RR = 0.39, 95%CI 0.15: 1.01, *p* = 0.0509). In addition, there was no heterogeneity among included studies, as shown in Fig. 7.

**Fig. 8:**
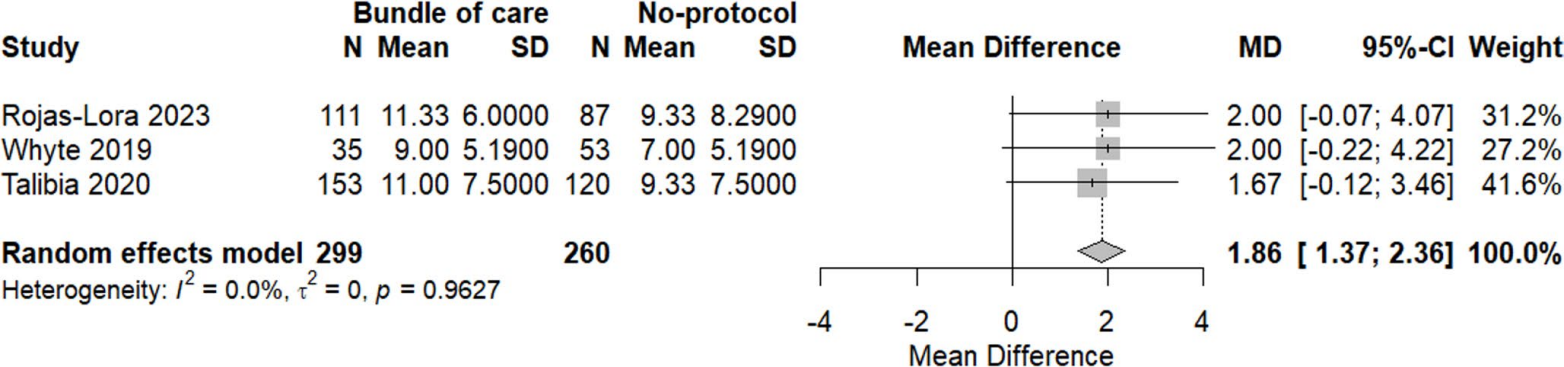
Meta-analysis of drain stays

Regarding drain stay, a meta-analysis was done; there was a significantly lower duration in the bundle of care group (MD = 1.89, 95% CI: 1.37; 2.36, *p* = 0.0038). No heterogeneity was found, as seen in Fig. 8.

**Fig. 9:**
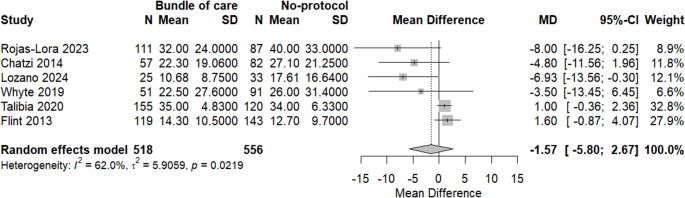
A meta-analysis of hospital stay duration between both groups

A meta-analysis of six studies was done to assess the difference in hospital stay duration between applying a bundle of care and not applying it; there was no significant difference in hospital stay in the bundle group with MD = −1.57 (95% CI: −5.80; 2.67, *p* = 0.3855). Moreover, there was a significant heterogeneity among included studies, with I^2^ = 62%, as shown in Fig. 9.

**Fig. 10:**
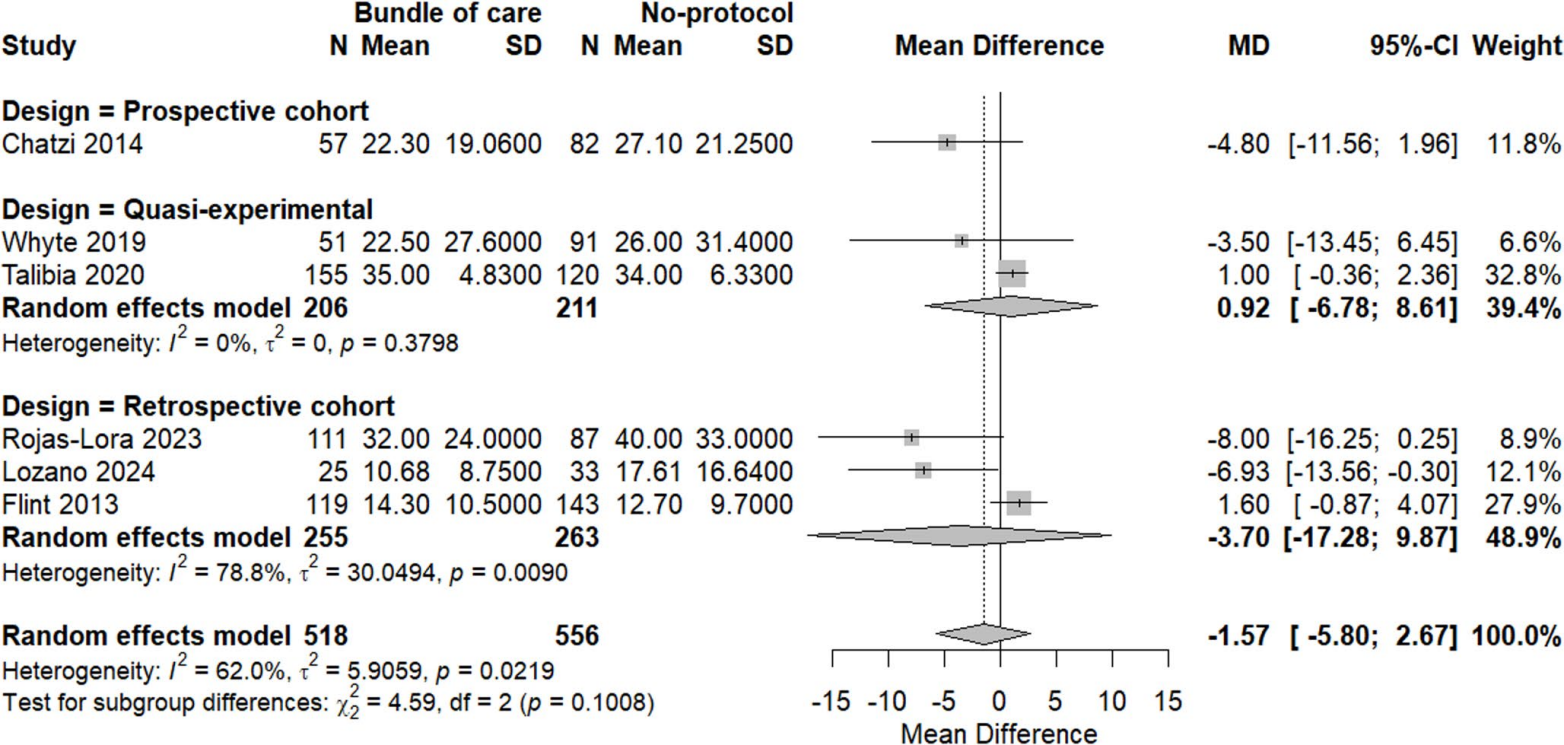
Subgrouping analysis of hospital stays based on study design

Subgrouping analysis revealed that heterogeneity was resolved among quasi-experimental studies, while retrospective studies showed significant heterogeneity without any significant change in the context of the results. There was no significant difference between subgroups, as shown in Fig. 10.

## Discussion

Infection rates associated with shunts have been growing rapidly in recent years. Sometimes, shunts are a lifesaving procedure to aid in decreasing intracranial pressure; the literature reports incidence rates varying between 3.1% and 11.4 per 1,000 catheter-days, indicating a continuous need to make the procedure safer for patients [28, 29]. This systematic review and meta-analysis evaluated the impact of implementing care bundles on outcomes related to EVDs.

The results of the present meta-analysis show a notable decrease in EVDI incidence through the use of care bundles, which aligns with earlier research emphasizing the effectiveness of such standardized preventive strategies. Choo et al. reported a reduction in EVDI rates from 16.7% to 4.0% after implementing a care bundle based on guidelines from the Centers for Disease Control and Prevention [14]. Similarly, Lora et al. reported a 58% lower EVDI rate after implementing a bundle that emphasized CSF sampling minimization [5]. These findings support the protective effect of care bundles observed in the present meta-analysis.

Furthermore, there was no significant difference in the incidence of gram-negative or gram-positive bacterial infections associated with EVD between the care bundle and control groups in this study. Gram-negative organisms remained the most frequently isolated pathogens, in agreement with prior studies reporting a predominance of gram-negative bacteria. In contrast, Jamjoom et al. reported that Gram-positive organisms were the most frequent in their study [30]. 

Our results did not reveal a statistically significant difference in mortality rates or length of hospital stay between patients managed with a care bundle and those receiving standard care in the context of EVD. While care bundles have been shown in some studies to improve compliance with best practices and reduce certain complications, such as catheter-related infections, their impact on hard clinical outcomes, including mortality and hospital stay, remains controversial [5, 11, 31]. Numerous studies have reported that implementing a care bundle reduces EVD-related infection rates, but there is no significant effect on mortality or length of stay, aligning with our current findings [5, 15, 21]. These findings suggest that while care bundles are beneficial in standardizing care and reducing preventable adverse events, broader clinical outcomes such as mortality may be influenced by multifactorial elements beyond the scope of bundle-based interventions. More high-quality, controlled studies are needed to determine whether additional factors, such as timing, patient severity, or institutional resources, modulate the effectiveness of care bundles on long-term outcomes.

The effectiveness of care bundles depends on proper implementation, staff adherence, and institutional support. Several studies have highlighted the importance of multidisciplinary collaboration, staff education, and strict adherence to standardized protocols in achieving sustained reductions in infection rates [22, 32]. 

## Limitations

This meta-analysis has several limitations. First, the “care bundle” components were not uniform: each study implemented a slightly different set of practices, and specific measures (e.g., use of antibiotic-impregnated catheters, frequency of CSF sampling, use of prophylactic antibiotics) were often poorly detailed. Protocols for EVD care varied greatly between hospitals, and adherence also differed. This inconsistency makes it difficult to generalize findings; a bundle that was effective in one setting may not have the same impact in another if the components or compliance differ. In addition, definitions of EVD infection varied, with some studies requiring positive CSF cultures with clinical signs, while others included any positive culture or used different diagnostic time frames. This contributed to the substantial heterogeneity observed among the included studies. Although only bundles containing more than three components were included, methodological variability remained significant. Additionally, the care bundle had no statistically significant impact on patient-centered outcomes such as mortality or hospital length of stay. Across the aggregated data, implementing EVD care bundles did not measurably reduce death rates or shorten hospitalization time compared to standard care. This is even though EVD infections themselves are associated with higher mortality and prolonged hospital stay in individual studies. This may reflect the influence of multiple confounding clinical factors unrelated to EVD infection alone.

## Conclusion

This meta-analysis supports the use of care bundles in lowering EVDIs. The general trend shows that care bundles have a preventive action against infections. Still unclear whether there are no improvements in terms of mortality and hospital stay, which emphasizes the necessity of more research.

Future research should focus on enhancing the implementation of standardized elements of the bundle of care and investigating other interventions to improve overall clinical outcomes.

## Supplementary Information

Below is the link to the electronic supplementary material.

Supplementary Material 1 (DOCX 111 KB)

## Data Availability

No datasets were generated or analysed during the current study.

## Abbreviations

AICs: Antibiotic-impregnated catheters
CI: Confidence interval
CSF: Cerebrospinal fluid
MD: Mean differences
EVDs: External ventricular drains
EVDI: External ventricular drain-related infection
RCTs: Randomized controlled trials

## References

[1] Chau CYC, Craven CL, Rubiano AM, Adams H, Tülü S, Czosnyka M et al (2019) The evolution of the role of external ventricular drainage in traumatic brain injury. J Clin Med. 10.3390/jcm8091422 31509945 10.3390/jcm8091422 PMC6780113

[2] Hallenberger TJ, Tharmagulasingam T, Licci M, Mariani L, Guzman R, Soleman J (2024) Management of external ventricular drain: to wean or not to wean? Acta Neurochir (Wien). 10.1007/s00701-024-06166-z 38954061 10.1007/s00701-024-06166-z PMC11219415

[3] Lewis A, Czeisler BM, Lord AS (2017) Variations in strategies to prevent Ventriculostomy-Related infections. Neurohospitalist 7:15–23. 10.1177/1941874416663281 28042365 10.1177/1941874416663281 PMC5167094

[4] Dasic D, Hanna SJ, Bojanic S, Kerr RSC (2006) External ventricular drain infection: the effect of a strict protocol on infection rates and a review of the literature. Br J Neurosurg 20:296–300. 10.1080/02688690600999901 17129877 10.1080/02688690600999901

[5] Rojas-Lora M, Corral L, Zabaleta-Carvajal I, López-Ojeda P, Fuentes-Mila V, Romera-Peregrina I et al (2023) External ventriculostomy-associated infection reduction after updating a care bundle. Ann Clin Microbiol Antimicrob. 10.1186/s12941-023-00612-z 37454149 10.1186/s12941-023-00612-z PMC10349458

[6] De Andrade AYT, Canicoba ARB, Oliveira RA, Gnatta JR, de Brito Poveda V (2025) Risk factors for infection associated with the use of external ventricular drainage: a systematic review with meta-analysis. J Hosp Infect 162:368–376. 10.1016/j.jhin.2024.07.004 39032570 10.1016/j.jhin.2024.07.004

[7] Zhou J, Zhong Y, Li X, Li H, Wang J, Yang S et al (2023) Risk Factors for External Ventricular Drainage-Related Infection: A Systematic Review and Meta-analysis. Neurol Clin Pract 13(4):e200156. 10.1212/CPJ.0000000000200156 37529300 10.1212/CPJ.0000000000200156 PMC10238084

[8] Konstantelias Α, Vardakas K, Polyzos K, Tansarli G, Falagas M (2015) Antimicrobial-impregnated and -coated shunt catheters for prevention of infections in patients with hydrocephalus: a systematic review and meta-analysis. J Neurosurg 122:1–17. 10.3171/2014.12.JNS14908 25768831 10.3171/2014.12.JNS14908

[9] Cui Z, Wang B, Zhong Z, Sun Y, Sun Q, Yang G et al (2015) 1;43(7):e23-32 Impact of antibiotic- and silver-impregnated external ventricular drains on the risk of infections: A systematic review and meta-analysis. Am J Infect Control. 10.1016/j.ajic.2015.03.015 10.1016/j.ajic.2015.03.015 25934064

[10] Blacker SN, Prabhakar H, Moreton EO, Burbridge M, Dunn L, Gouker LN et al (2025) Effect of bundled care on external ventricular drain infections: A systematic review and Meta-analysis. J Neurosurg Anesthesiol 17. 10.1097/ANA.0000000000001071 10.1097/ANA.0000000000001071 41243982

[11] Kubilay Z, Amini S, Fauerbach L, Archibald L, Friedman W, Layon A (2012) Decreasing ventricular infections through the use of a ventriculostomy placement bundle: experience at a single institution. J Neurosurg 118. 10.3171/2012.11.JNS121336 10.3171/2012.11.JNS121336 23259820

[12] Page MJ, McKenzie JE, Bossuyt PM, Boutron I, Hoffmann TC, Mulrow CD et al (2021) The PRISMA 2020 statement: an updated guideline for reporting systematic reviews. BMJ 372:n71. 10.1136/bmj.n71 33782057 10.1136/bmj.n71 PMC8005924

[13] Ates N, Kafadar A, Aygun G, Yildirim A (2020) Usage of a bundle application process in decreasing ventriculoperitoneal shunt infections. Turk Neurosurg 30:550–556. 10.5137/1019-5149.JTN.26864-19.3 31736037 10.5137/1019-5149.JTN.26864-19.3

[14] Choo YH, Shim Y, Kim H, Goh HY, Kim SJ, Kim EJ et al (2023) Significant reduction in external ventricular drain-related infections after introducing a novel bundle protocol: a before and after trial. J Korean Med Sci. 10.3346/jkms.2023.38.e386 38147836 10.3346/jkms.2023.38.e386 PMC10752748

[15] Flint AC, Rao VA, Renda NC, Faigeles BS, Lasman TE, Sheridan W (2013) A simple protocol to prevent external ventricular drain infections. Neurosurgery 72:993–999. 10.1227/NEU.0b013e31828e8dfd 23467249 10.1227/NEU.0b013e31828e8dfd

[16] Camacho EF, Boszczowski Í, Freire MP, Pinto FCG, Guimaraes T, Teixeira MJ et al (2013) Impact of an educational intervention implanted in a neurological intensive care unit on rates of infection related to external ventricular drains. PLoS One. 10.1371/journal.pone.0050708 23390486 10.1371/journal.pone.0050708 PMC3563649

[17] Rivero-Garvía M, Márquez-Rivas J, Jiménez-Mejías ME, Neth O, Rueda-Torres AB (2011) Reduction in external ventricular drain infection rate. Impact of a minimal handling protocol and antibiotic-impregnated catheters. Acta Neurochir (Wien) 153:647–651. 10.1007/s00701-010-0905-1 21170556 10.1007/s00701-010-0905-1

[18] Hong B, Apedjinou A, Heissler HE, Chaib H, Lang JM, Al-Afif S et al Effect of a bundle approach on external ventricular drain-related infection n.d. 10.1007/s00701-020-04698-8/Published 10.1007/s00701-020-04698-8 33427989

[19] Whyte C, Alhasani H, Caplan R, Tully AP (2020) Impact of an external ventricular drain bundle and limited duration antibiotic prophylaxis on drain-related infections and antibiotic resistance. Clin Neurol Neurosurg. 10.1016/j.clineuro.2019.105641 31869626 10.1016/j.clineuro.2019.105641

[20] Sweeney J, Zyck S, Tovar-Spinoza Z, Krishnamurthy S, Chin L, Bodman A (2019) Evidence-based perioperative protocol for ventriculoperitoneal shunt infection reduction at a single institution. World Neurosurg 128:e814–e822. 10.1016/j.wneu.2019.04.261 31078805 10.1016/j.wneu.2019.04.261

[21] Talibi SS, Silva AHD, Afshari FT, Hodson J, Roberts SAG, Oppenheim B et al (2020) The implementation of an external ventricular drain care bundle to reduce infection rates. Br J Neurosurg 34:181–6. 10.1080/02688697.2020.1725436 32046516 10.1080/02688697.2020.1725436

[22] Chatzi M, Karvouniaris M, Makris D, Tsimitrea E, Gatos C, Tasiou A et al (2014) Bundle of measures for external cerebral ventricular drainage-associated ventriculitis. Crit Care Med 42:66–73. 10.1097/CCM.0b013e31829a70a5 23982025 10.1097/CCM.0b013e31829a70a5

[23] Dasgupta D, D’Antona L, Cat DA, Toma AK, Curtis C, Watkins LD et al (2019) Simulation workshops as an adjunct to perioperative care bundles in the management of external ventricular drains: improving surgical technique and reducing infection. J Neurosurg 131:1620–4. 10.3171/2018.5.JNS172881 30497209 10.3171/2018.5.JNS172881

[24] Reiter LA, Taylor OL, Jatta M, Plaster SE, Cannon JD, McDaniel BL et al (2023) Reducing external ventricular drain associated ventriculitis: an improvement project in a level 1 trauma center. Am J Infect Control 51:644–51. 10.1016/j.ajic.2022.08.029 36116678 10.1016/j.ajic.2022.08.029

[25] Lozano M, Wang AS, Siddiqi I, Dy O, Ko K, Sweiss R et al (2024) Extraventricular Drain Care Bundle Decreases Cerebrospinal Fluid Infection Rates Associated With Extraventricular Drain-Related Procedures and Systemic Infection. Cureus. 10.7759/cureus.52440 10.7759/cureus.52440 PMC10871024 38371086

[26] Ciorba VCGAMLCD (2015) Reducing external drainage-related cerebrospinal fluid infections through implementation of a multidisciplinary protocol: experience in a paediatric hospital 26499427

[27] Korinek AM, Reina M, Boch AL, Rivera AO, De Bels D, Puybasset L (2005) Prevention of external ventricular drain - related ventriculitis. Acta Neurochir (Wien) 147:39–46. 10.1007/s00701-004-0416-z 15565481 10.1007/s00701-004-0416-z

[28] Sweid A, Weinberg JH, Abbas R, El Naamani K, Tjoumakaris S, Wamsley C et al (2021) Predictors of ventriculostomy infection in a large single-center cohort. J Neurosurg 134:1218–25. 10.3171/2020.2.JNS192051 32276249 10.3171/2020.2.JNS192051

[29] Ramanan M, Lipman J, Shorr A, Shankar A (2015) A meta-analysis of ventriculostomy-associated cerebrospinal fluid infections. BMC Infect Dis 15:3. 10.1186/s12879-014-0712-z 25567583 10.1186/s12879-014-0712-z PMC4300210

[30] Jamjoom AAB, Joannides AJ, Poon MTC, Chari A, Zaben M, Abdulla MAH et al (2018) Prospective, multicentre study of external ventricular drainage-related infections in the UK and Ireland. J Neurol Neurosurg Psychiatry 89:120–126. 10.1136/jnnp-2017-316415 29070645 10.1136/jnnp-2017-316415 PMC5800336

[31] Blot K, Bergs J, Vogelaers D, Blot S, Vandijck D (2014) Prevention of central line–associated bloodstream infections through quality improvement interventions: a systematic review and meta-analysis. Clin Infect Dis 59:96–105. 10.1093/cid/ciu239 24723276 10.1093/cid/ciu239 PMC4305144

[32] Gilhooly D, Green SA, McCann C, Black N, Moonesinghe SR (2019) Barriers and facilitators to the successful development, implementation and evaluation of care bundles in acute care in hospital: a scoping review. Implement Sci 14:47. 10.1186/s13012-019-0894-2 31060625 10.1186/s13012-019-0894-2 PMC6501296

