# Supplementary material for: Is there a way to reduce the infection associated with external ventricular shunts? A systematic review and meta-analysis of the recent bundle of care
Source: Neurosurg Rev. 2026 Jan 19;49(1):133. doi: 10.1007/s10143-025-04029-4 (PMC12812772; doi:10.1007/s10143-025-04029-4)
Supplement: Supplementary file 1 — Supplementary Material 1 (DOCX 111 KB) [file 10143_2025_4029_MOESM1_ESM.docx]

# Table (1): Baseline Characteristics, bundle components and quality of included studies for EVD bundle

| Study ID | Groups | Country | Age mean (SD) | Gender m\f | Bundle components | Quality |
| --- | --- | --- | --- | --- | --- | --- |
| Rojas-Lora 2023 | Pre-protocol | Spain | 53 (14) | 38/49 | Outdated protocol | Good |
|  | Post-protocol |  | 57 (15) | 52/59 | Updated protocol |  |
| Lozano 2024 | Pre-intervention | USA | 48.45 (20.11) | 24/9 | Four  standardization procedures, associated checklists, and monitoring requirements | Good |
|  | Post-intervention |  | 48.24 (17.59) | 14/11 |  |  |
| Dasgupta 2018 | 1^st^ Post-intervention group (2016) | UK | 54 (15) | 21/22 | Silver-impregnated catheter, Prophylactic antibiotics, Minimal shaving with clippers only and preparation with alcoholic chlorhexidine before draping | Good |
|  | 2^nd^ Post-intervention group (2017) |  | 54 (18) | 28/20 |  |  |
| Dasic 2006 | Pre-protocol | UK | 53 | 27/14 | Outdated protocol | Fair |
|  | Post-protocol |  | 56 | 32/22 | Updated protocol |  |
| Ciorba 2015 | Pre-protocol | Italy |  |  | Outdated protocol | Fair |
|  | Post-protocol |  |  |  | Updated protocol |  |
| Reiter DNP 2023 | pre-intervention | USA |  |  | strict asepsis with full barrier precautions for all procedures  involving EVD manipulation; minimizing EVD handling; stopping  routine culturing; use of do not inject labeling; standardized  EVD dressing and dressing changes only when compromised; and  development of an EVD transport protocol. Additionally, routine  zeroing of the intracranial pressure (ICP) monitoring transducer was  discontinued and replaced with zeroing at the time of insertion, with  system disconnection, and when the ICP value does not match the  clinical picture (eg, troubleshooting). Cap, mask, and sterile gloves  are used for the zeroing procedure and the procedure is completed  with replacement of a new sterile dead-end cap on the transducer  stopcock port | Poor |
|  | Post-intervention |  |  |  |  |  |
| Rivero-Garvía 2010 | group1 | Spain |  |  | No minimal handling protocol or antibiotic impregnated catheter | Poor |
|  | group 2 |  |  |  | Minimal handling protocol only |  |
|  | group 3 |  |  |  | Minimal handling protocol and antibiotic impregnated catheters |  |
| Whyte 2019 | Pre-intervention | USA | 55.6 (16.1) | 54/37 | Outdated protocol | Good |
|  | Post-intervention |  | 60.5 (17.3) | 30/24 | Updated protocol |  |
| Sweeney2019 | Pre-protocol | USA | 32.4 (27.6) | 120/106 | Outdated protocol | Good |
|  | Post-protocol |  | 29.7 (25.4) | 178/107 | Updated protocol |  |
| Talibi2020 | Pre-intervention | UK | 53.3 (15.75) | 69/51 | generic aseptic techniques | Good |
|  | Post-intervention |  | 55.6 (17.21) | 86/69 | hand-washing technique, use of surgical  theatre standard scrub and preparation, and cleaning of the  EVD access ports, together with gown, gloves and mask |  |
| Chatzi 2014 | Pre-intervention | Greece | 53.5 (15.47) | 51/31 | Outdated protocol | Good |
|  | Post-intervention |  | 50.3 (16.2) | 38/19 | 1) reeducation  of ICU personnel on issues of infection control related to external  cerebral ventricular drainage, 2) meticulous intraventricular catheter  handling, 3) cerebrospinal fluid sampling only when clinically  necessary, and 4) routine replacement of the drainage catheter on  the seventh drainage day if the catheter was still necessary |  |
| Ates 2019 | Pre-intervention | Turkey |  | 24/20 | Outdated protocol | Poor |
|  | Post-intervention |  |  | 25/13 | using 2%  chlorhexidine gluconate +70% alcohol for skin cleaning,  changing the dressing every 48 hours, and observing the  operating room |  |
| Camacho 2013 | Pre-intervention | Brazil | 49 (12.5) | 25/49 | Outdated protocol | Fair |
|  | Post-intervention |  | 48 (14.33) | 42/62 | Updated protocol |  |
| Korinek 2004 | Pre-protocol | France | 49 (16) | 79/52 | Outdated protocol | Good |
|  | Post-protocol |  | 44 (17) | 114/61 | Updated protocol |  |
| Flint2013 | Pre-protocol | USA | 61.33(17.22) | 47/96 | Outdated protocol | Fair |
|  | Post-protocol |  | 60.3(18.01) | 54/65 | Updated protocol |  |
| Hong2021 | Pre-intervention | Germany | 48.6 (21.6) | 77/64 | Outdated protocol | Fair |
|  | Post-intervention |  | 50.9(21.2) | 96/112 | Updated protocol |  |
| Choo2023 | Pre-protocol | South korea | 57 (11.83) | 42/42 | Outdated protocol | Good |
|  | Post-protocol |  | 59 (12) | 41/58 | Updated protocol |  |


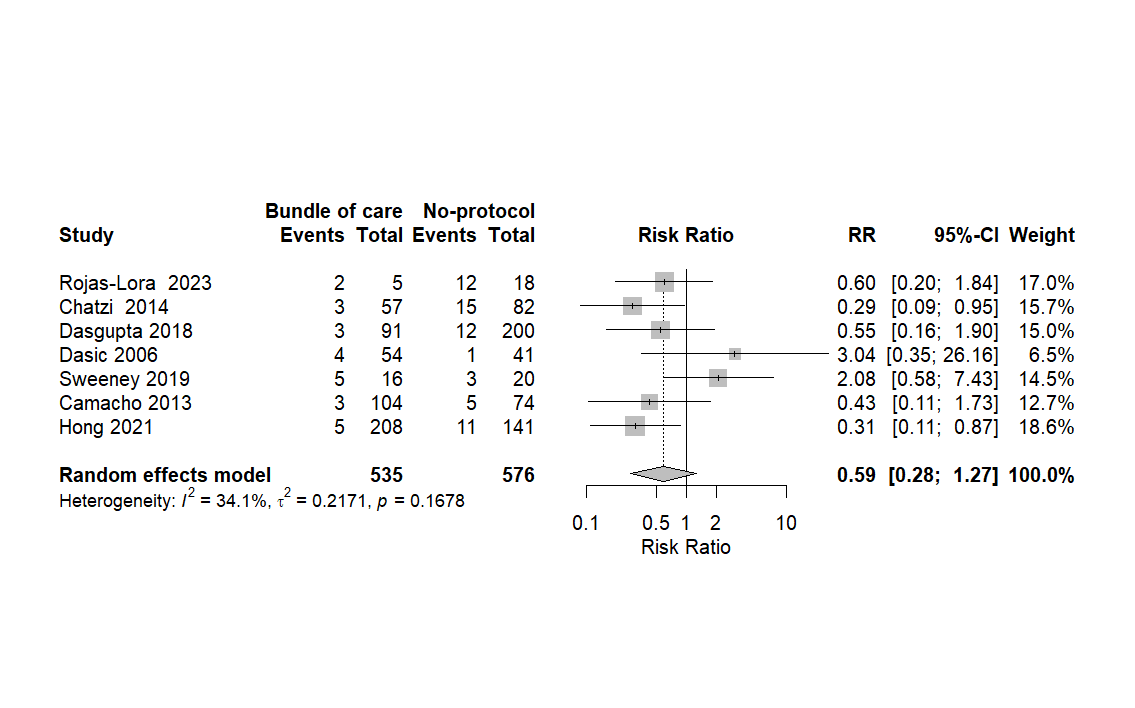


Figure S1: Forest plot of the incidence of gram-negative infection in both groups.


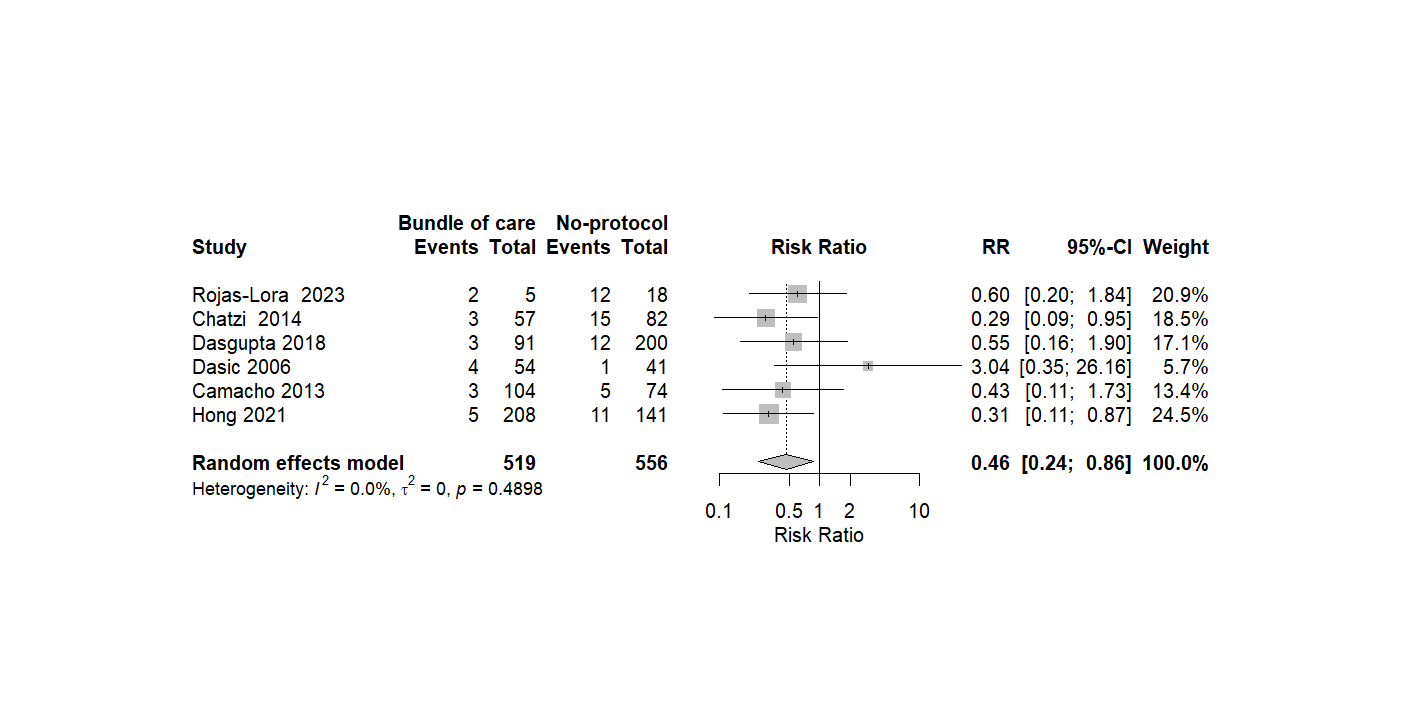


Figure S2: Sensitivity analysis of the incidence of gram-negative infection in both groups.


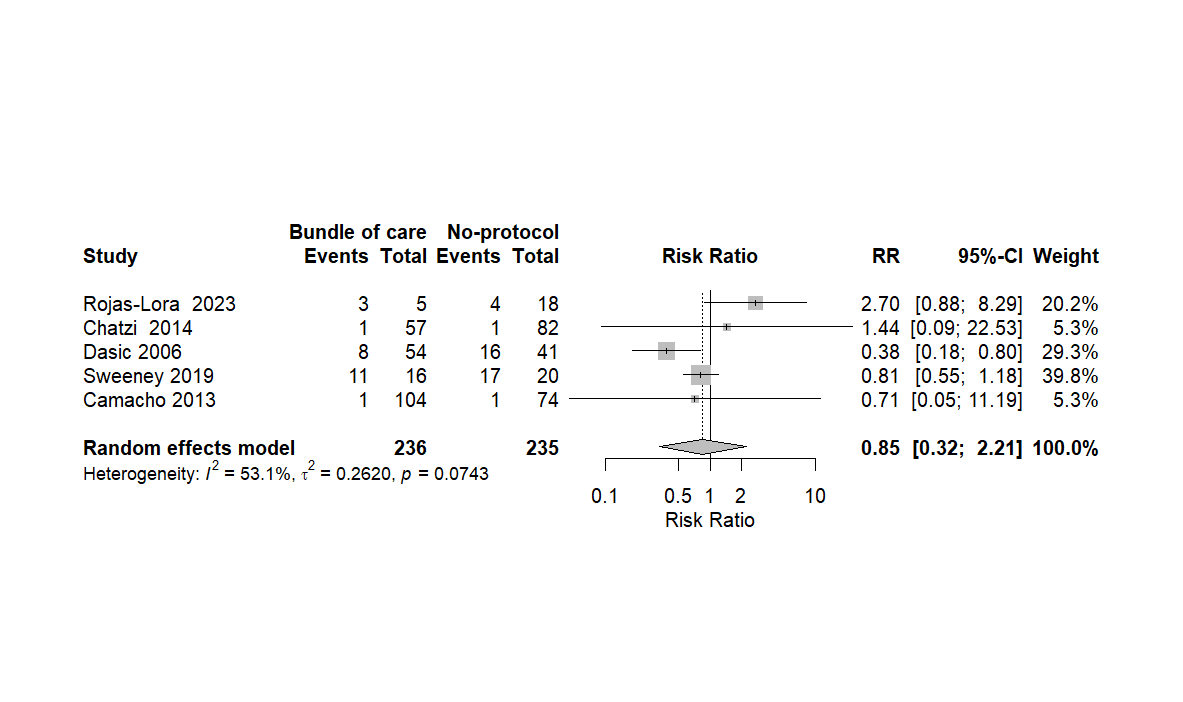


Figure S3: Forest plot of the incidence of gram-positive infection in both groups.


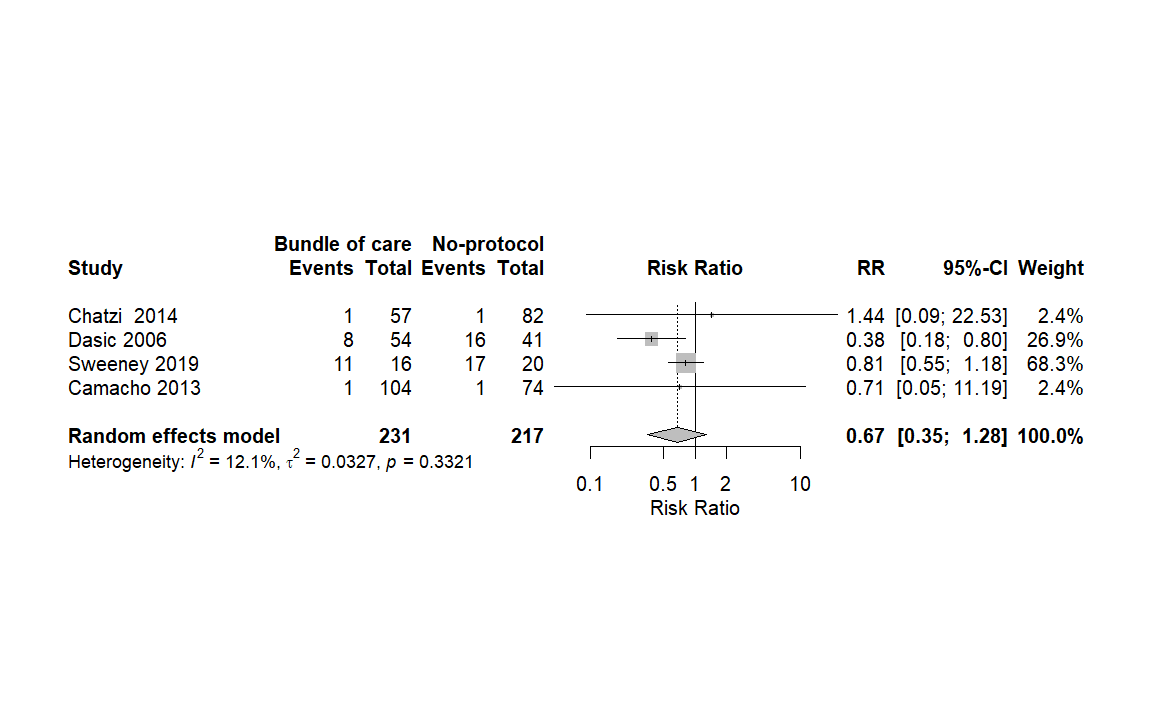


Figure S4: Sensitivity analysis of the incidence of gram-positive infection in both groups.
